# CGtag: complete genomics toolkit and annotation in a cloud-based Galaxy

**DOI:** 10.1186/2047-217X-3-1

**Published:** 2014-01-24

**Authors:** Saskia Hiltemann, Hailiang Mei, Mattias de Hollander, Ivo Palli, Peter van der Spek, Guido Jenster, Andrew Stubbs

**Affiliations:** 1 Department of Bioinformatics, Erasmus MC, Dr. Molewaterplein 5, 3015 GE Rotterdam, The Netherlands; 2 Department of Urology, Erasmus MC, Dr. Molewaterplein 5, 3015 GE Rotterdam, The Netherlands; 3 Netherlands Bioinformatics Center, NBIC, Geert Grooteplein 28, 6525 GA Nijmegen, The Netherlands; 4 Department of Microbial Ecology, Netherlands Institute of Ecology, NIOO-KNAW, Droevendaalsesteeg 10, 6708 PB Wageningen, The Netherlands

**Keywords:** Complete genomics, Next generation sequencing, Genetic variation, Pathogenic gene selection

## Abstract

**Background:**

Complete Genomics provides an open-source suite of command-line tools for the analysis of their CG-formatted mapped sequencing files. Determination of; for example, the functional impact of detected variants, requires annotation with various databases that often require command-line and/or programming experience; thus, limiting their use to the average research scientist. We have therefore implemented this CG toolkit, together with a number of annotation, visualisation and file manipulation tools in Galaxy called CGtag (Complete Genomics Toolkit and Annotation in a Cloud-based Galaxy).

**Findings:**

In order to provide research scientists with web-based, simple and accurate analytical and visualisation applications for the selection of candidate mutations from Complete Genomics data, we have implemented the open-source Complete Genomics tool set, CGATools, in Galaxy. In addition we implemented some of the most popular command-line annotation and visualisation tools to allow research scientists to select candidate pathological mutations (SNV, and indels). Furthermore, we have developed a cloud-based public Galaxy instance to host the CGtag toolkit and other associated modules.

**Conclusions:**

CGtag provides a user-friendly interface to all research scientists wishing to select candidate variants from CG or other next-generation sequencing platforms’ data. By using a cloud-based infrastructure, we can also assure sufficient and on-demand computation and storage resources to handle the analysis tasks. The tools are freely available for use from an NBIC/CTMM-TraIT (The Netherlands Bioinformatics Center/Center for Translational Molecular Medicine) cloud-based Galaxy instance, or can be installed to a local (production) Galaxy via the NBIC Galaxy tool shed.

## Findings

### Background

Complete Genomics (CG) supplies results for whole-genome next-generation sequencing (NGS) data mapped to a user-defined genome [1] and additional open-source tools [2] for further characterisation of the sequenced genomes. Whilst these tools are open-source and available for download and use on the command-line, they are not amenable for scientists to use from their desktop, and require scripting skills to link these tools together with other applications to successfully prioritise candidate pathogenic genes based on these NGS results. To address this issue, we implemented the Complete Genomics Analysis Toolkit (CGATools), including several functional annotation and visualisation tools in a cloud-enabled instance of Galaxy. Galaxy offers a web-based graphical user interface to command-line tools, and allows for the graphical construction of complex workflows; Galaxy will automatically keep track of the analysis history, and allows for easy sharing and publishing of data and/or workflows with other users [3-5]. Furthermore, Galaxy is an extensible platform, nearly any software tool may be integrated into Galaxy, and there is an active community of users and developers ensuring the latest tools are made available for use in Galaxy through the Galaxy tool shed.

This implementation of the CGATools in a Galaxy environment simplifies the analysis of genomes via the Galaxy GUI and the cloud resource ensures that sufficient computing power is available for the analysis. The inherent functionality in Galaxy of CGtag enables the creation of customisable user-defined workflows by the scientist and not only by the bioinformatician.

For large datasets, transfer to Galaxy via SFTP is available and recommended, but is still limited by the upload speed of the user’s internet connection, and can be a bottleneck in the analysis of large datasets.

### Variant detection

CGATools is an open-source project to provide tools for downstream analysis of Complete Genomics data, and may be downloaded from their repository [2]. These tools must be run from the command-line and are therefore, not accessible to all users. To remedy this, Complete Genomics also provide Galaxy tool wrappers for many of the CGAtools, which can be downloaded from the Main Galaxy tool repository (tool shed) [6]. However, these Galaxy tools still need to be installed on the users’ local (production) Galaxy instance before they can be utilised. We have now made these tools available on a public server [7], and have added Galaxy wrappers for those CGAtools that were not provided by Complete Genomics e.g. Junctions2Events, makeVCF (Table 1). The use of the CGAtools in Table 1 have previously been outlined [8], using a combination of ListVariants and TestVariants or CallDiff to determine candidate pathogenic single nucleotide variants (SNVs), indels and subs in a selected genome as compared with on or more reference genomes or as part of a trio based genetic analysis [8]. The VarFilter may be used to select those variants which have a high confidence based on the underlying sequence reads as specified as VQHIGH, and the SNPDiff tool can then be used to determine concordance of the NGS results with those of an orthogonal SNV detection platform such as an Affymetrix or Illumina SNP array. The JunctionDiff and Junction2Events tools are used to select fusion events and candidate fusion genes based on quality of the discordant reads used to detect the structural variation event [9].

**Table 1:** Overview of CGTag tools available in NBIC/CTMM-TraIT Galaxy and the NBIC tool shed

**Function**	**Tool**	**Description**
Variant detection	CGATools ListVariants	Lists the non-redundant set of small variations found in an arbitrary number of genomes.
Variant detection	CGATools TestVariants	Determine which variants are found in which genomes given the results of ListVariants.
Variant detection	CGATools CallDiff	Compares two variant files to determine where and how the genomes differ.
Variant detection	CGATools VarFilter	Copies the input varfile or masterVar file, applying filters.
Variant detection	CGATools JunctionDiff	Reports difference between junction calls of CG junction files.
Variant detection	CGATools Junctions2Events	Groups and annotate related junctions.
Quality control	CGATools SNPDiff	Compares genotype calls to CG variant files.
File merge	CGATools Join	Merge two tab-delimited files based on equal field or overlapping regions.
Sequence retrieval	CGATools DecodeCRR	Retrieve sequences from a CRR file for a given range of a chromosome.
File conversion	CGATools mkVCF	Converts CG variant and/or junction files to VCF.
File conversion	CGATools generateMasterVar	Converts a varfile to a on-line-per-locus format.
File conversion	CGATools fasta-2-crr	Converts fasta sequences into a single reference crr file.
File conversion	CGATools crr-2-fasta	Converts crr file to fasta sequence.
File conversion	TestVariants2VCF	CG community tool. Converts output of the TestVariants tool to VCF.
Annotation	ANNOVAR	Functional annotation of genetic variants from high-throughput sequencing data.
Annotation	MutationAssessor	Functional impact of protein mutations.
Annotation	Condel	CONsensus DELeteriousness score of missense SNVs.
Visualisation	CG Circos plots	Create CG-style tumour, normal and somatic plots.
Visualisation	Integrative plot	Create circos plot from CG and SNParray data.
Visualisation	Generic genomic data plotter	Plot any type of numerical genomic data using GNUplot.
File manipulation	Filter columns	Filter tab-delimited files based on column contents.
File manipulation	Add/remove chr prefix	adds or removes chr prefix from chromosome column.
File manipulation	Column select	Extract and/or rearrange columns in tab-delimited file.
File manipulation	Sort chromosomal position	Sort a tab-delimited file by chromosomal position.
File manipulation	Strip header	Remove header from files.
File manipulation	File concatenation	Concatenate 2 files (e.g. for restoring header).

### Functional annotation tools

To provide users with enhanced filtering capabilities, we have integrated several command-line annotation tools in this NBIC/CTMM-TraIT Galaxy instance. ANNOVAR [10] is a command-line tool used to functionally annotate genetic variants. We provide a Galaxy tool wrapper for ANNOVAR. This tool will take a list of variants as input and provide gene and amino acid change annotation, SIFT scores, PolyPhen scores, LRT scores, MutationTaster scores, PhyloP conservation scores, GERP++ conservation scores, DGV variant annotation, dbSNP identifiers, 1000 Genomes Project allele frequencies, NHLBI-ESP 6500 exome project allele frequencies, and other information. We have implemented this tool to accept VCF (v4) files, Complete Genomics varfiles or CG-derived tab-separated files using the CG 0-based half-open coordinate system, or lastly, the standard ANNOVAR input format consisting of tab-separated lists of variants using the 1-based coordinate system. This tool will output the original file columns, followed by additional ANNOVAR columns. The ANNOVAR code itself is not included in the tool shed repository, but instructions on how to obtain a license and the subsequent manual installation of the tool are included in the readme of the Galaxy tool shed repository. We obtained permission to offer ANNOVAR on our public Galaxy server, so the tool can be previewed there. To supplement ANNOVAR, Condel (CONsensus DELeteriousness) [11] has been included to calculate the deleterious score associated of missense SNVs and the impact of non-synonymous SNVs on protein function. Condel integrates the outputs of two tools: SIFT and Polyphen2, to calculate a weighted average of the scores (WAS) of these tools. Condel can optionally incorporate the output of a third tool, MutationAssessor, which is also included in this Galaxy instance. Mutation Assessor [12] is a web-based tool providing predictions of the functional impact of amino-acid substitutions in proteins, such as mutations discovered in cancer or missense polymorphisms. The MutationAssessor database is accessed through a REST API. In order not to overload the server, queries are limited to 3 per second, so when dealing with a long list of variants, some pre-filtering is recommended. The functional annotation provided by ANNOVAR, including the addition of multiple versions of dbSNP, the variants provided by Complete Genomics Public data from unrelated individuals only [13] and 31 genomes from Huvariome [14], are available in this Galaxy instance. Huvariome provides the user with additional whole genome variant calls for those regions which are difficult to sequence and can retrieve the weighted allele frequency for each base in the human genome [14].

### Visualisation tools

**Figure 1:**
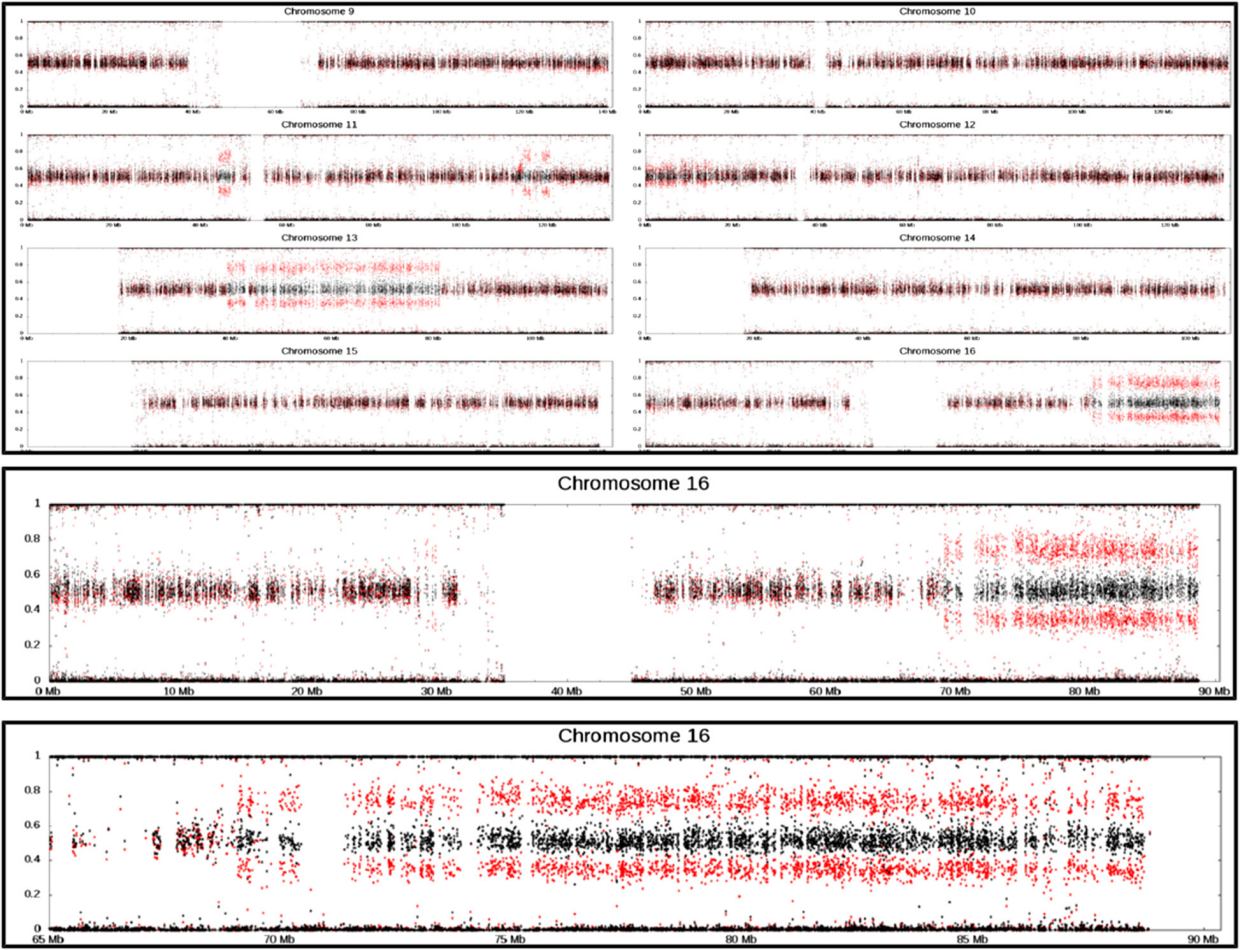
**Generic genomic data plotting tool.** Output from our generic genomic data plotter used to plot B-allele frequency from Illumina 1M SNParray data. Plot with two tracks; tumour (red) and normal (black). Output can be (top) a whole genome overview (shown here in part), or (middle) a single chromosome, or (bottom) a subregion of a chromosome defined by the user (here chr16, 60MB-end). Many parameters such as the colour and sizes of the data points may be adjusted by the user as required.

A generic genomic data plotter tool based on GNUplot is available, which takes as input, a tab-delimited file of format chr–start-end–value, and will output either a single chromosome plot, an overview of all chromosome plots in a single image, or a sub-region of a chromosome defined by the user. Additionally, the tool has the option of plotting input from a second file in the same image, which is useful for tumour-normal comparison (Figure 1). B-allele frequency (BAF) is used to determine whether the structural variation junction is homo- or heterozygous. When the data is in the right format, the generic plotter tool can be used to visualise the BAF, and we have also implemented a plot tool to display allele frequencies directly from a CG masterVar file, again with the capability of displaying single-chromosome plots, all chromosomes in a single image, or custom defined regions (Figure 1). The current Complete Genomics analysis pipeline (CGAP v2.5) delivers Circos [15] visualisations with each genome that is sequenced and the code used to generate these images have been made freely available for download [16]. We have modified this code and implemented Galaxy tools to allow for the generation of these images for samples sequenced on earlier CG analysis pipelines (before v2.0), that utilise the junctions file, masterVar file, CNV details and CNV segments files to generate the standard CG Circos report.

**Figure 2:**
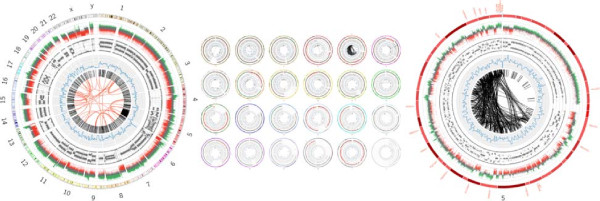
**Circos integrative plot tool.** Circos plots for (left) whole genome, (middle) overview or all chromosomes in single images, and (right) for a single chromosome. Each chromosome is represented in the outer ring and then from outer to inner rings represent copy number variation (with regions of gain depicted in green and loss in red), B-allele frequency, SNP density and the intra- and interchromosomal rearrangements are on the inside and depicted in black and red lines, respectively. Impacted genes track (red gene symbols) are displayed outside the outer chromosome ring and only on the single chromosome plot.

To support fusion gene analysis we have created a custom Circos tool which uses CG files, CG junctions file and CG varfile for NGS, and the results from SNP arrays analysis, specifically the B-allele frequency (BAF) and copy number variation (CNV) files. The output is either a whole-genome plot, per-chromosome plots, a single image containing all the per-chromosome plots together, or a plot of a custom region defined by the user (e.g., a plot showing just chromosomes 3, 5, and X, or a plot showing a specific range within a single chromosome). Additionally the user can select an “impacted genes” track for the per-chromosome plots, which will print the names of the genes impacted by SV events along the outer edge of the image (Figure 2). This custom Circos script is capable of using fusion gene detection results generated from the Illumina platform with the fusion genes detected by an application such as FusionMap [17], and which are reported in custom FusionMap report format, a tab-delimited file similar to that delivered by Complete Genomics.

In addition to these tools within Galaxy, structural variation files processed using CGtag may be exported to our previously described fusion gene prioritisation tool, iFUSE [18] to identify candidate fusion genes and display their representative DNA, RNA and protein sequence.

### Auxiliary tools

Our suite of tools also includes several auxiliary tools supplied by CG but not available from the Galaxy tool shed which offer the user several file format conversion tools (Table 1) that enable users to connect the output from the CGATools analysis to other analytical or annotation workflows by means of standard file formats (e.g., FASTA, VCF). In addition a number of file formatting tools are also included, such as removing of headers from files (required by some tools), adding removing of a chr prefix to a column of a file (i.e., chrX vs. X), concatenation of files, and extracting and rearranging of columns, to help facilitate the flow of data from one tool to the next.

### CLOUD implementation

NBIC Galaxy is hosted at a high performance computing (HPC) cloud system operated by SURFsara [19]. This HPC cloud consists of 19 fast servers with 608 CPUs and almost 5TB of memory. The NBIC Galaxy that operates in this HPC cloud is implemented using the Cloudman framework [20] and its adapted version supports the OpenNebula Cloud environment. The advantage of using the Cloudman framework to build NBIC Galaxy is mainly two-fold, firstly Cloudman provides a set of complete scripts to automatically install tools and datasets on a virtual machine image. The installed tools include the Galaxy system itself and all its dependencies. These dependencies include webserver (nginx), database (postgres), cluster job scheduler (SGE), and common NGS tools, such as bowtie, BWA, samtools, and so forth. The installed datasets include most of the common reference genomes (hg18, hg19, mm9, etc) and their tool-specific index files. Thus, the end product of running Cloudman installation script is a fully functional NBIC Galaxy system operating in the HPC Cloud.

The second contribution of Cloudman to our NBIC Galaxy system is its ability to set up a flexible virtual cluster and ability to provide auto-scaling support. The previous NBIC Galaxy was hosted on a dedicate physical server with rather limit resources (4 CPU, 32G memory). Due to this resource limitation, our NBIC Galaxy was never promoted to be a real data analysis server to handle the production level of NGS datasets. On the other hand, because of the sporadic nature of user access, the server was mostly on idle during its 2-year lifespan. Moving to Cloud resolved both issues. The current NBIC Galaxy operates on top of a virtual cluster. This virtual cluster contains one head node and a number of worker nodes. These nodes are all virtual machines that are built using the machine image generated by the Cloudman script. During minimal usage, the cluster will only contain one head node. Once a significant load occurs due to training courses or production level data analysis, the virtual cluster can automatically scale itself upwards. More worker nodes will be added dynamically to this virtual cluster to boost the capacity of NBIC Galaxy. Once the load decreases, the virtual cluster can scale down again to operate with only a limited number of nodes.

The use of shared resources does have drawback as well. We have experienced a more obvious I/O bottleneck in the cloud-based NBIC Galaxy compared to the previous system that ran in a physical machine. In the HPC Cloud, storage is provided through a network file system (NFS) instead of a local hard disk. When more concurrent Cloud users are using the Cloud resource, we observe the extra job time caused by I/O delays. However, we argue that this issue is far outweighed by the benefit of having a dynamic virtual cluster support to the NBIC Galaxy.

## Availability and requirements

**Project Name:** CGtag: Complete Genomics Toolkit and Annotation in a Cloud-based Galaxy **Project home page:**http://galaxy.ctmm-trait.nl**Operating system:** Linux (Galaxy and CGtag)**Programming language:** Python (Galaxy and CGtag), R (CGtag), Bash (CGTag) **Other requirements:** Circos [15], GNUplot [21], Complete Genomics open source Toolkit [2] and dependencies therein); see documentation for a comprehensive list of optional dependencies, based on workflow requirements.**License:** GPL v3Restrictions to use by non-academics: ANNOVAR license must be obtained before it can be used.Galaxy resources: published page: http://galaxy.ctmm-trait.nl/u/saskia-hiltemann/p/cgtag Links to tool shed repositories:annovar: http://toolshed.nbic.nl/view/saskia-hiltemann/annovar cgatools: http://toolshed.nbic.nl/view/saskia-hiltemann/cgatools\_v17 circos plotters: http://toolshed.nbic.nl/view/saskia-hiltemann/cg\_circos\_plots condel: http://toolshed.nbic.nl/view/saskia-hiltemann/condel file manipulation tools: http://toolshed.nbic.nl/view/saskia-hiltemann/file\_manipulation generic genomic data plotter: http://toolshed.nbic.nl/view/saskia-hiltemann/genomic\_data\_plotter mutation assessor: http://toolshed.nbic.nl/view/saskia-hiltemann/mutation\_assessor NOTE: these tools can be installed to both Cloudman Galaxy instances or non-Cloudman Galaxy instances alike (via the tool shed or manually from the command line).

## Availability and supporting data

All tools described, as well as example data, are available from the NBIC/CTMM-TraIT Galaxy server (http://galaxy.ctmm-trait.nl) and the NBIC Galaxy tool shed (http://toolshed.nbic.nl).

## Abbreviations

BAF: B-Allele frequency; CG: Complete genomics; CGATools: Complete genomics analysis tools; CGtag: Complete genomics toolkit and annotation in a cloud-based galaxy; NBIC: The Netherlands Bioinformatics Center; NFS: Network file system; NGS: Next generation sequencing; SNV: Single nucleotide variation; SV: Structural variation.

## Competing interests

The authors declare that they have no competing interests.

## Authors’ contributions

SH, GJ, HM and AS contributed to the design and coordination of CGtag and manuscript preparation. SH, MdH, IP and HM contributed to implementing CGtag. SH, GJ, PvdS and AS contributed to testing of CGtag. MdH and HM implemented Galaxy Cloudman for the SURFsara/Big-grid HPC cloud. All authors read and approved the final manuscript.

## Note from the Editors

This paper is part of the GigaScience Galaxy series. We will be hosting some of the computational resources of these papers on our GigaGalaxy server (http://galaxy.cbiit.cuhk.edu.hk).

## References

[B1] Drmanac R Sparks AB Callow MJ Halpern AL Burns NL Kermani BG Carnevali P Nazarenko I Nilsen GB Yeung G Dahl F Fernandez A Staker B Pant KP Baccash J Borcherding AP Brownley A Cedeno R Chen L Chernikoff D Cheung A Chirita R Curson B Ebert JC Hacker CR Hartlage R Hauser B Huang S Jiang Y Karpinchyk V Human genome sequencing using unchained base reads on self-assembling DNA nanoarrays Science 2010 327 5961 78 81 10.1126/science.1181498 19892942

[B2] CGATools [http://cgatools.sourceforge.net/]

[B3] Goecks J Nekrutenko A Taylor J The Galaxy Team Galaxy: a comprehensive approach for supporting accessible, reproducible, and transparent computational research in the life sciences Genome Biol 2010 11 8 R86 10.1186/gb-2010-11-8-r86 20738864 PMC2945788

[B4] Blankenberg D Von Kuster G Coraor N Ananda G Lazarus R Mangan M Nekrutenko A Taylor J Galaxy: a web-based genome analysis tool for experimentalists Curr Protoc Mol Biol 2010 89 19.10.1 19.10.21 10.1002/0471142727.mb1910s89 PMC4264107 20069535

[B5] Giardine B Riemer C Hardison RC Burhans R Elnitski L Shah P Zhang Y Blankenberg D Albert I Taylor J Miller W Kent WJ Nekrutenko A Galaxy: a platform for interactive large-scale genome analysis Genome Res 2005 15 10 1451 1455 10.1101/gr.4086505 16169926 PMC1240089

[B6] Main Galaxy Toolshed [http://toolshed.g2.bx.psu.edu/]

[B7] NBIC CTMM-TraIT Cloud-based Galaxy [http://galaxy.ctmm-trait.nl]

[B8] Florisson JMG Verkerk AJMH Huigh D Hoogeboom AJM Swagemakers S Kremer A Heijsman D Lequin MH Mathijssen IMJ van der Spek PJ Boston type craniosynostosis: report of a second mutation in MSX2 Am J Med Genet A 2013 161 2626 2633 23949913 10.1002/ajmg.a.36126

[B9] Hiltemann SD McClellan EA van Nijnatten J Horsman S Palli I Teles Alves I Hartjes T Trapman J van der Spek P Jenster G Stubbs A iFUSE: integrated fusion gene explorer Bioinformatics 2013 29 13 1700 1701 10.1093/bioinformatics/btt252 23661695

[B10] Wang K Li M Hakonarson H ANNOVAR: functional annotation of genetic variants from high-throughput sequencing data Nucleic Acids Res 2010 38 16 e164 10.1093/nar/gkq603 20601685 PMC2938201

[B11] González-Pérez A López-Bigas N Improving the assessment of the outcome of nonsynonymous SNVs with a consensus deleteriousness score condel Am J Hum Genet 2011 88 4 440 449 10.1016/j.ajhg.2011.03.004 21457909 PMC3071923

[B12] Reva B Antipin Y Sander C Predicting the functional impact of protein mutations application to cancer genomics Nucleic Acids Res 2011 39 17 e118 10.1093/nar/gkr407 21727090 PMC3177186

[B13] Complete Genomics ftp [ftp.completegenomics.com, ftp2.completegenomics.com]

[B14] Stubbs A McClellan EA Horsman S Hiltemann SD Palli I Nouwens S Koning AH Hoogland F Reumers J Heijsman D Swagemakers S Kremer A Meijerink J Lambrechts D van der Spek PJ Huvariome: a web server resource of whole genome next-generation sequencing allelic frequencies to aid in pathological candidate gene selection J Clin Bioinformatics 2012 2 19 10.1186/2043-9113-2-19 PMC3549785 23164068

[B15] Circos Circular Visualisation [http://circos.ca]

[B16] CG circos scripts [ftp://ftp.completegenomics.com/ToolRepository/CompleteCircosPackage.zip]

[B17] Liu K Juan T Fang F Newman M Hoeck W Ge1 H FusionMap: detecting fusion genes from next-generation sequencing data at base-pair resolution Bioinformatics 1922 27 14 10.1093/bioinformatics/btr310 21593131

[B18] iFUSE integrated fusion gene explorer [http://ifuse.erasmusmc.nl] 10.1093/bioinformatics/btt252 23661695

[B19] SURF Sara HPC Cloud [http://www.surfsara.nl/systems/hpc-cloud]

[B20] Afgan E Baker D Coraor N Chapman B Nekrutenko A Taylor J Galaxy CloudMan delivering cloud compute clusters BMC Bioinformatics 2010 11 Suppl12 S4 21210983 10.1186/1471-2105-11-S12-S4 PMC3040530

[B21] GNUPlot [http://www.gnuplot.info]

